# Preliminary hematological reference values and morphology of blood cells in farmed sex-reversed male Nile tilapia (*Oreochromis niloticus*) from Southern Thailand

**DOI:** 10.14202/vetworld.2026.1773-1784

**Published:** 2026-04-30

**Authors:** Kannawee Swangneat, Duangkamon Phromduang, Tran Nhat Thang, Weerapong Laovechprasit, Pornchai Pornpanom

**Affiliations:** 1 Akkhraratchakumari Veterinary College, Walailak University, Nakhon Si Thammarat, 80160, Thailand; 2 One Health Research Center, Walailak University, Nakhon Si Thammarat, 80160, Thailand; 3 Faculty of Animal Science and Veterinary Medicine, Thai Nguyen University of Agriculture and Forestry, Thai Nguyen, 24119, Vietnam; 4 Department of Pathology, College of Veterinary Medicine, University of Georgia, Athens, GA 30602, USA; 5 Informatics Innovation Center of Excellence, Walailak University, Nakhon Si Thammarat, 80160, Thailand

**Keywords:** blood cell morphology, hematological parameters, Nile tilapia, piscine hematology, reference values, sex-reversed males, Southern Thailand, white blood cells

## Abstract

**Background and Aim:**

Hematological assessment is a fundamental tool for evaluating fish health, yet its application in teleost species remains constrained by the reliance on manual methods because of the presence of nucleated blood cells. Consequently, accurate interpretation of blood cell morphology and the availability of reliable reference data are critical for diagnostic standardization. This study aimed to characterize blood cell morphology and establish preliminary hematological values in farmed sex-reversed male Nile tilapia (*Oreochromis niloticus*) (NT) raised in Southern Thailand, thereby supporting laboratory competency and aquaculture health management.

**Materials and Methods:**

Blood samples were initially collected from 46 sex-reversed male NT. Samples were evaluated for quality, and those containing blast cells, apoptotic cells, hemolysis, or suspected parasitic inclusions were excluded. A total of 14 high-quality samples were included for detailed analysis. Blood smears were prepared using the push technique and stained with Wright’s stain. Hematological parameters, including packed cell volume (PCV), hemoglobin (Hb) concentration, red blood cells (RBC) count, white blood cell (WBC) count, and differential leukocyte counts, were determined using standard manual methods. Morphological and morphometric evaluations of blood cells were conducted using light microscopy and image analysis software.

**Results:**

Erythrocytes, leukocytes, and thrombocytes were identified, with leukocytes classified into neutrophils, basophils, eosinophils, lymphocytes, and monocytes. Lymphocytes were the predominant leukocyte type, followed by neutrophils, whereas eosinophils and basophils were rarely observed. Monocytes were the largest cells (mean diameter: 15.56 ± 1.8 µm), while lymphocytes were the smallest (6.89 ± 0.99 µm). Hematological analysis revealed PCV ranging from 0.23 to 0.45 L/L, Hb concentration from 76 to 119 g/L, RBC counts from 0.94 to 2.72 × 10¹²/L, and WBC counts from 4.17 to 23.01 × 10^9^/L. An *Anaplasmataceae*-like inclusion body was observed in one sample, although confirmatory diagnosis was not performed.

**Conclusion:**

This study provides preliminary hematological and morphological reference data for farmed NT, contributing to improved diagnostic accuracy and laboratory standardization. The findings support fish health assessment, facilitate future research, and enhance sustainable aquaculture practices and food security.

## INTRODUCTION

Nile tilapia (*Oreochromis niloticus*) (NT) is a teleost fish of major economic importance, characterized by its wide geographic distribution, high global market demand, rapid growth, ease of cultivation, and tolerance to diverse environmental conditions [1]. Farmed NT has been established in Thailand for over 40 years, during which the country has contributed substantially to global production. In 2024, NT production reached approximately 275,958 tons, valued at USD 385 million and representing 58.93% of national freshwater aquaculture output [2]. Several production systems are used for NT culture, including earthen ponds, cages, and concrete ponds, supporting food security and livelihoods in rural and urban communities.

Sex control represents an effective strategy for improving production [3], as males grow faster and exhibit better feed conversion efficiency than females [4, 5], making mono-sex male NT culture a widely adopted practice in Thailand. However, fish health and animal welfare remain key concerns in tilapia aquaculture, especially under intensive culture systems [6], highlighting the need for sustainable management practices that balance productivity with animal welfare and environmental responsibility.

In aquaculture, hematological assessments, particularly complete blood counts (CBCs), are commonly applied to monitor fish health and welfare [7], and have gained importance with increasing concern over aquatic pollution [8, 9]. Routine CBCs in fish are mainly carried out manually because all blood cells are nucleated, which limits the use of automated hematology analyzers [10]. Automated WBCs (WBC) counts may be inaccurate due to incomplete lysis of nucleated erythrocytes (RBCs) and thrombocytes [11]. Recent evidence suggests that automated analyzers can provide reliable results for RBC and WBC counts; however, these results should be validated through manual analyses [12].

Manual CBCs require trained expertise because laboratory results may vary depending on stress levels, blood collection techniques, storage conditions, laboratory procedures, and accuracy of cell identification [10, 13–15]. Additionally, manual CBCs enable veterinary technicians to detect blood parasites simultaneously with WBC differential counts. Common piscine blood parasites include *Anaplasmataceae* spp., *Cryptobia* (*Trypanoplasma*) spp., *Trypanosoma* spp., hemogregarines, and piroplasmids [16, 17]. Of these, *Anaplasmataceae* spp. and *Trypanosoma* spp. have been found in NT [17–20], although no documented cases have been reported in Thailand. Because both differential counts and parasite detection require a strong understanding of blood cell and parasite morphology, investigations that provide scientific reference data are essential for strengthening laboratory competency.

Additionally, interpretation of CBCs in fish is challenging due to the absence of standardized reference intervals, which vary across species, age, sex, nutritional status, temperature, and water quality [21–25]. In Thailand, hematologic values have been reported for several fish species and are widely used as indicators of health and environmental conditions, including Asian seabass (*Lates calcarifer*) [26] and Siamese tiger fish (*Datnioides microlepis*) [27]. For NT, previous studies have reported hematological values under different environmental conditions or bacterial challenges [28, 29]. However, blood cell morphological descriptions remain incomplete and insufficient [24, 30], and a wide range of hematological reference intervals has been reported [31]. Furthermore, locally applicable hematologic reference data for clinical services are still lacking.

The present study was designed to comprehensively investigate the hematological characteristics of farmed sex-reversed male NT raised in Southern Thailand, with a particular focus on generating baseline data directly applicable in clinical and laboratory settings. Specifically, the study aimed to (i) describe in detail the morphology of peripheral blood cells, including RBCs, WBCs, and thrombocytes, using standardized hematological techniques; (ii) perform morphometric evaluation of different blood cell types to establish quantitative cellular dimensions; and (iii) determine preliminary hematological values, including packed cell volume (PCV), hemoglobin (Hb) concentration, RBC count, WBC count, and differential leukocyte counts. In addition, the study sought to identify potential hematological abnormalities and detect any blood-borne inclusions suggestive of parasitic or infectious agents. By integrating qualitative and quantitative hematological analyses under controlled laboratory conditions, this research aimed to provide scientifically validated reference data that can enhance diagnostic accuracy, strengthen laboratory competency in piscine hematology, and support health monitoring and disease management in aquaculture systems. Ultimately, the findings are intended to contribute to the standardization of hematological evaluation in NT and to promote sustainable aquaculture practices and food security.

## MATERIALS AND METHODS

### Ethical approval

The experimental protocol was reviewed and approved by the Walailak University Institutional Animal Care and Use Committee (WU-IACUC), Walailak University, Nakhon Si Thammarat, Thailand (Approval No. WU-ACUC-68002). All procedures involving NT were conducted in accordance with institutional guidelines for the care and use of animals in research and were designed to minimize pain, distress, and unnecessary handling. Fish used in this study were obtained from commercial farms during routine production and were handled only for blood collection. Before sampling, fish were anesthetized by immersion in a clove oil solution until loss of equilibrium and cessation of opercular movement, and sampling was performed promptly under humane conditions by trained personnel. During the procedure, fish were monitored for adequate anesthetic depth based on absence of response to external stimuli. No surgical intervention or experimental disease challenge was performed, and no fish were subjected to prolonged restraint or intentionally induced suffering. Blood collection was limited to the minimum volume required for hematological analysis, and all efforts were made to reduce stress associated with capture, handling, transport, and sampling. Therefore, the study complied with accepted ethical standards for the humane use of aquatic animals in research.

### Study period and location

This study was conducted from June to August 2025 in Southern Thailand. Blood samples were collected from farmed sex-reversed NT raised in Nakhon Si Thammarat (8°37′ N, 99°53′ E; n = 31) and Surat Thani (9°5′ N, 99°12′ E; n = 15). Blood collection and anesthesia were performed at the Center for Scientific and Technological Equipment (B3), Walailak University (8.64′ N, 99.89′ E). Hematological analyses were conducted at the Laboratory of Veterinary Clinical Pathology, Akkhraratchakumari Veterinary College, Walailak University (8.64′ N, 99.89′ E).

### Fish and sampling sites

Sex-reversed male NT with body weights of 500–1,000 g were obtained from commercial farms in Nakhon Si Thammarat (8°37′ N, 99°53′ E; n = 31) and Surat Thani (9°5′ N, 99°12′ E; n = 15), Southern Thailand, between June 2025 and August 2025 using convenience sampling. Farms used semi-intensive earthen pond systems with commercial pellet feed. Data on water quality parameters (temperature, pH, dissolved oxygen, ammonia, and nitrite) were unavailable. The fish were approximately 7 months old and considered market-ready adults.

### Fish anesthesia and blood sample collection

Fish were anesthetized by immersion in a 30 ppm clove oil solution (prepared by diluting 6 mL of 10% clove oil stock in 20 L aerated water) until loss of equilibrium and cessation of opercular movement (typically 3–5 min). Blood (approximately 0.5–1.0 mL) was collected from the caudal vasculature using a 24-gauge needle attached to a 1 mL syringe. Blood was immediately transferred into tubes containing ethylenediaminetetraacetic acid (EDTA; Quetainer™, Cangzhou Fukang Medical Supplies, Hebei, China) at a final concentration of approximately 1.8 mg/mL.

EDTA was selected as the anticoagulant based on its common use in piscine hematology for cell counting; however, the literature indicates potential erythrocyte swelling with EDTA in some teleosts [32], and heparin may preserve morphology better in certain species.

Samples were grouped as: market-transported (Group I, n = 14), fresh-caught with 24-h iced storage (Group II, n = 15), and fresh-caught with immediate analysis (Group III, n = 17).

Blood smear preparation and staining: Three blood smears were prepared per fish using the push-pull technique on clean glass slides. Smears were air-dried and stained with Wright’s stain (Wright eosin methylene blue solution, Merck KGaA, Darmstadt, Germany) as follows: the dried smears were immersed with Wright’s stain for 6 min (this step also served as the fixation step [33]; no prior methanol fixation was required), after which buffer was added and allowed to stand for 8 min. Then, the stained blood smears were washed in running tap water.

### Hematological analysis in fishes

Hb concentration was measured using an automated analyzer (URIT-300Plus, URIT Medical Electronic, Dhaka, Bangladesh) on whole blood. Other hematologic parameters were manually evaluated by veterinary technicians following standard methods [34–36]. PCV was determined by microhematocrit centrifugation (14,000 × *g* for 5 min).

Total RBC and WBC counts were performed manually after 1:200 dilution in Natt and Herrick’s solution (in-house preparation: 0.10 g methyl violet 2B, 3.88 g sodium chloride, 2.50 g sodium sulfate, 2.91 g disodium hydrogen phosphate dodecahydrate, 0.25 g potassium phosphate, 7.50 mL formalin 37%, distilled water to 1,000 mL; pH adjusted to 7.3) [37]. Counts were made in a Neubauer hemocytometer chamber (BOECO GermanyTM, Boeckel + CO, Hamburg, Germany) under 400× magnification.

WBC counts were corrected for thrombocytes using the formula:

Corrected WBC count (×10^9^/L) = Uncorrected WBC (×10^9^/L) × 100 / (Thrombocytes per 100 WBC + 100)

Differential leukocyte counts and thrombocyte enumeration were performed on 100 leukocytes per smear at 1,000× magnification. All counts were conducted by two trained veterinary technicians blinded to group assignment.

Absolute counts of leukocyte types were calculated as:

Absolute number (×10^9^/L) = Relative percentage × Corrected WBC count (×10^9^/L) / 100

RBC indices were computed as follows:

MCV (fL) = PCV (%) × 10 / RBC (×10^6^/µL)

MCH (pg) = Hb (g/dL) × 10 / RBC (×10^6^/µL)

MCHC (g/dL) = Hb (g/dL) × 100 / PCV (%)

### Blood smear examination and morphometric measurement of blood cells

Smears were screened at 400× magnification for 100 fields to detect morphological abnormalities, parasites, or artifacts, followed by detailed examination at 1,000× magnification for another 100 fields. Only smears from Group III fish without blast cells, apoptotic cells, or significant karyorrhexis were included for morphology and morphometric analysis, and for calculation of preliminary hematological values. Images were captured using an Olympus BX43 microscope equipped with a DP27 camera and CellSens software (version 1.18, Olympus, Tokyo, Japan). Morphometry was performed on 100 randomly selected cells per type (except basophils, which were limited to 10 cells due to rarity) in the monolayer area. Cell length, width, area (erythrocytes), or diameter (leukocytes) were measured using calibrated software tools.

### Statistical analysis

Data were analyzed using descriptive statistics (mean ± standard deviation, minimum–maximum, and median). Histograms were generated using the *ggplot2* package in R version 4.5.2 [38] to visualize distributions. Given the small final sample size (n = 14), formal reference intervals were not calculated according to the American Society for Veterinary Clinical Pathology recommendations (ideally n ≥ 20–40 for non-parametric methods; n ≥ 120 preferred for robust estimation) [39]. Outliers were screened visually using the *boxplot* package in R version 4.5.2 [38] but were retained unless biologically implausible. No inferential statistics were applied between groups due to exclusion criteria and sample size limitations.

## RESULTS

### Blood smear examination and exclusion criteria

**Figure 1:**
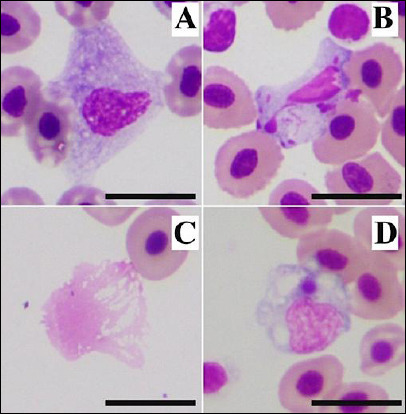
Abnormal cells were found in farmed Nile tilapia; these cells were used as exclusion criteria for blood cell morphology and hematology analyses. (A) Blast cell/transformed mature cell: A large cell with abundant dark-purple cytoplasm and a round to oval nucleus; (B) apoptotic cell: A leukocyte-like cell containing a karyorrhectic nucleus, observed in prolonged-storage samples; (C) smudge (basket) cell found in the same prolonged-storage samples; and (D) monocyte engulfing an Anaplasmataceae-like inclusion body. Wright’s stain. Scale bar = 10 µm.

Blood smear examination of 46 sex-reversed male NT revealed blast cells/transformed mature cells (Figure 1A) and apoptotic cells (Figure 1B) in all samples from Group I (market-transported fishes) and Group II (fresh-caught with prolonged-storage), respectively. Additionally, all blood smears from Group II contained several basket cells (Figure 1C). Thus, these 29 samples were excluded from hematologic analysis and morphological studies.

The main findings of this study were observed in Group III (fresh-caught with immediate hematological analysis). In this group, two samples showed hemolytic plasma and one sample revealed an intracytoplasmic inclusion body in a monocyte (Figure 1D). This structure morphologically resembled morulae of bacteria belonging to the order Rickettsiales, family Anaplasmataceae. However, diagnostic confirmation was not performed.

### Morphology of blood cells

Within the remaining 14 fish from Group III, without clinical signs or external lesions, examination of blood smears revealed the morphology of RBCs, WBCs, and thrombocytes. Notably, some fish contained a few karyorrhectic cells and immature cells.

**Figure 2:**
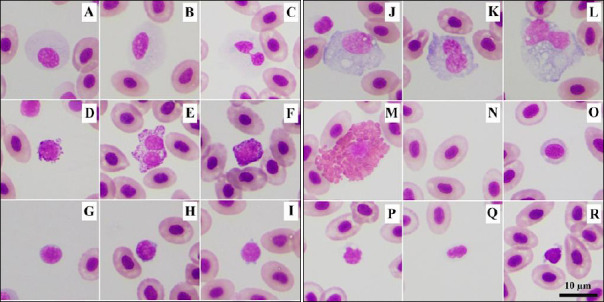
Blood cells of farmed Nile tilapia. (A, B) Neutrophils with round to oval nuclei; (C) occasionally, a bi-lobed nucleus was found; (D–F) basophils containing small basophilic granules; (G–I) lymphocytes with scant light-blue cytoplasm; (J–L) monocytes with abundant gray-blue cytoplasm containing numerous vacuoles; (M) eosinophil containing large, round eosinophilic granules; (N) mature erythrocyte; (O) immature erythrocyte; (P–Q) thrombocytes with clear cytoplasm containing small vacuoles; and (R) occasionally dark gray-blue cytoplasm. Wright’s stain.

The types of WBCs identified in NT raised in Southern Thailand included neutrophils, basophils, eosinophils, lymphocytes, and monocytes (Figures 2A–M).

Neutrophils were round cells with abundant clear cytoplasm (with some slightly light basophilic areas) and an eccentric round to oval nucleus, occasionally exhibiting a bi-lobed shape (Figure 2C). Granulation of neutrophils was not visible. Basophils were small, round cells with densely packed basophilic granules. The nucleus was round (Figures 2D and F), bi-lobed (Figure 2E), and usually obscured by these granules (Figure 2F), although it was occasionally clearly visible.

Lymphocytes were small, round cells with a narrow rim of light-blue cytoplasm. The nucleus was round (Figure 2G) to indented (Figure 2H) and eccentrically positioned. Occasionally, cytoplasmic blebs were observed (Figure 2I). Lymphocytes were the smallest WBCs, with a mean diameter of 6.89 ± 0.99 µm.

Monocytes were the largest cells, with a mean diameter of 15.56 ± 1.8 µm. They were round, oval, or irregular in shape and had abundant deep gray-blue cytoplasm, often containing vacuoles. The nucleus was round to oval (Figure 2J), kidney-shaped (Figure 2K), or irregularly shaped (Figure 2L).

Eosinophils, rarely found in NT, were round cells with a round to oval nucleus. Their cytoplasm contained large, round to oval eosinophilic granules (Figure 2M). Eosinophils were not found in the monolayer area and were detected only after examination of the entire smear.

Mature erythrocytes of varying sizes (Figure 2N) were ellipsoidal, nucleated cells with smooth eosinophilic cytoplasm and a centrally positioned oval nucleus. The mean cell length was 10.35 ± 0.85 µm, and the mean width was 7.03 ± 0.60 µm (Table 1). Immature erythrocytes with polychromatophilic cytoplasm were also found (Figure 2O).

**Table 1:** Morphometry of blood cells in farmed sex-reversed male Nile tilapia from Nakhon Si Thammarat, Southern Thailand.

Blood cells	Unit	Mean ± Standard deviation	Minimum–Maximum
Erythrocytes (n = 100)			
Length	µm	10.35 ± 0.85	8.65–11.99
Width	µm	7.03 ± 0.60	5.52–8.28
Area	µm²	62.92 ± 6.31	45.49–82.29
Diameter of leukocytes			
Neutrophils (n = 100)	µm	11.71 ± 1.15	9.26–14.71
Basophils (n = 10)	µm	8.58 ± 1.38	6.90–10.67
Lymphocytes (n = 100)	µm	6.89 ± 0.99	4.31–9.83
Monocytes (n = 100)	µm	15.56 ± 1.81	10.53–21.11

Thrombocytes were small, round (Figure 2P) or oval-shaped cells (Figure 2Q) with a condensed nucleus and clear cytoplasm that sometimes contained vacuoles. Occasionally, deep gray-blue cytoplasm was observed (Figure 2R).

### Morphometry of blood cells

Table 1 presents the morphometric measurements of blood cells in farmed sex-reversed male NT from Southern Thailand.

### Hematological values

**Figure 3:**
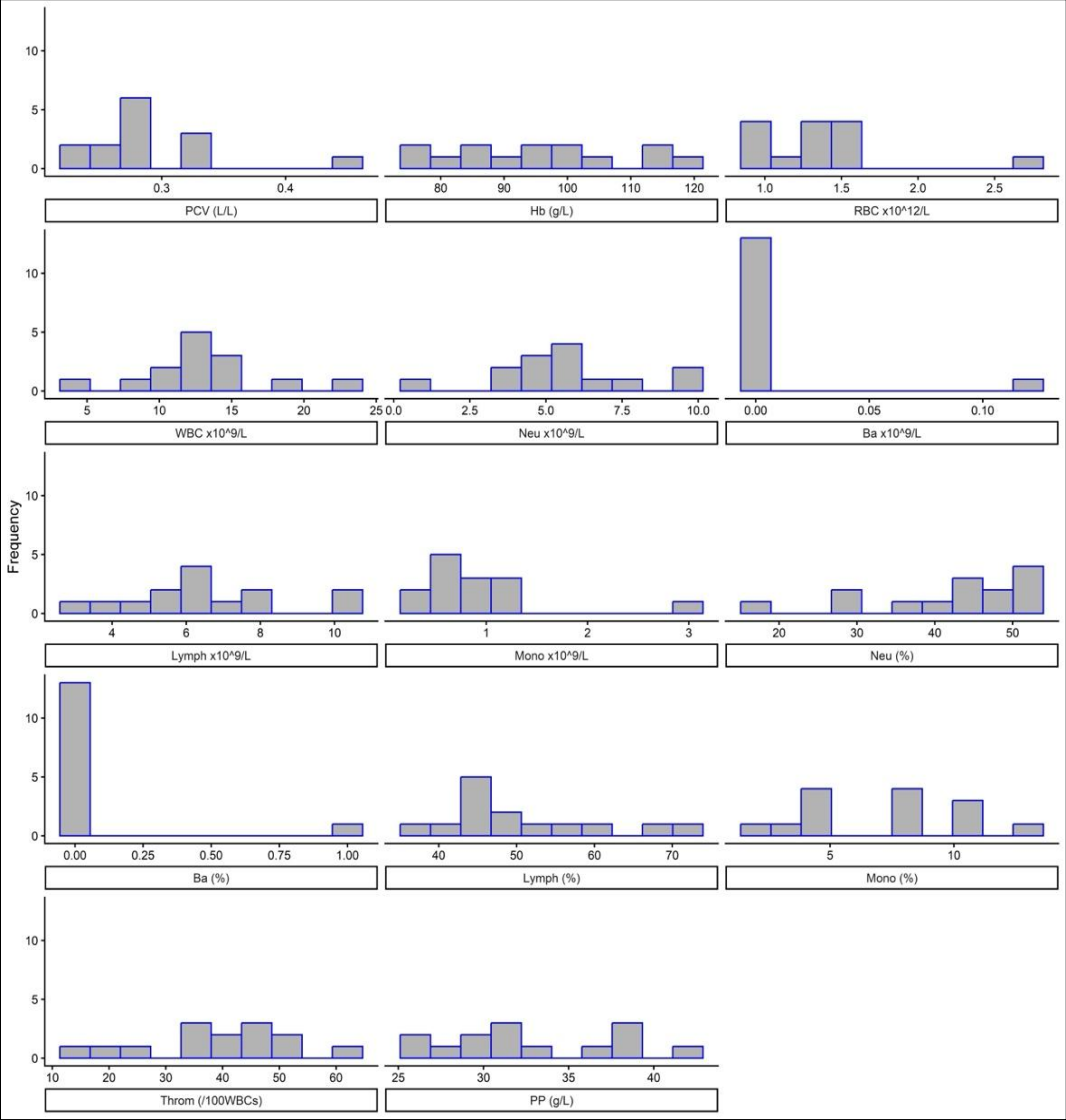
Histograms showing the distribution of hematological values from farmed Nile tilapia. The X-axis represents the measured values, whereas the Y-axis represents the frequency.

Individual hematologic values and their ranges are presented in Table 2, with distributions illustrated by histograms (Figure 3).

**Table 2:** Hematologic values of farmed sex-reversed male Nile tilapia (n = 14).

Parameters	SI unit	Ascending values	Mean ± Standard deviation	Median	Minimum–Maximum
Pack cell volume	L/L	0.23, 0.24, 0.26, 0.26, 0.27, 0.27, 0.27, 0.27, 0.28, 0.28, 0.32, 0.32, 0.33, 0.45	0.29 ± 0.05	0.27	0.23–0.45
Hemoblogin	g/L	76, 78, 80, 85, 87, 92, 97, 97, 98, 100, 104, 113, 114, 119	95.57 ± 13.66	97.00	76.00–119.00
Red blood cells	×10^12^/L	0.94, 1.01, 1.02, 1.03, 1.12, 1.25, 1.30, 1.33, 1.41, 1.49, 1.51, 1.52, 1.60, 2.72	1.37 ± 0.45	1.32	0.94–2.72
Mean corpuscular volume	fL	162.50, 165.44, 175.08, 185.43, 191.49, 207.69, 210.53, 236.45, 244.68, 248.12, 251.12, 256.00, 262.14, 268.66	218.95 ± 37.84	223.49	162.50–268.66
Mean corpuscular hemoglobin	pg	41.54, 60.53, 60.63, 64.24, 66.92, 69.50, 76.77, 76.85, 77.67, 80.85, 83.20, 84.58, 89.47, 89.69	73.03 ± 13.37	76.81	41.54–89.69
Mean corpuscular hemoglobin concentration	g/dL	25.11, 28.75, 29.63, 31.48, 32.22, 32.50, 32.50, 33.04, 34.64, 35.71, 36.06, 36.30, 37.31, 43.85	33.51 ± 4.45	32.77	25.11–43.85
White blood cells	×10^9^/L	4.17, 9.21, 9.71, 10.54, 11.65, 11.85, 11.92, 11.98, 13.34, 14.23, 14.77, 15.63, 18.10, 23.01	12.86 ± 4.40	11.95	4.17–23.01
Neutrophils	×10^9^/L	0.71, 3.44, 3.90, 4.43, 4.66, 4.79, 5.39, 5.72, 5.74, 6.06, 6.26, 7.97, 9.23, 9.66	5.57 ± 2.33	5.56	0.71–9.66
Eosinophils	×10^9^/L	0, 0, 0, 0, 0, 0, 0, 0, 0, 0, 0, 0, 0, 0	0.00 ± 0.00	0.00	0.00–0.00
Basophils	×10^9^/L	0, 0, 0, 0, 0, 0, 0, 0, 0, 0, 0, 0, 0, 0.12	0.01 ± 0.03	0.00	0.00–0.12
Lymphocytes	×10^9^/L	3.00, 3.41, 4.27, 5.13, 5.13, 5.99, 6.11, 6.41, 6.54, 7.11, 7.54, 8.15, 10.04, 10.35	6.37 ± 2.19	6.26	3.00–10.35
Monocytes	×10^9^/L	0.30, 0.43, 0.46, 0.47, 0.53, 0.60, 0.72, 0.78, 0.95, 1.01, 1.07, 1.19, 1.25, 2.99	0.91 ± 0.67	0.75	0.30–2.99
Neutrophils	%	17, 29, 30, 37, 42, 43, 44, 45, 48, 48, 51, 51, 52, 52	42.07 ± 10.42	44.50	17.00–52.00
Eosinophils	%	0, 0, 0, 0, 0, 0, 0, 0, 0, 0, 0, 0, 0, 0	0.00 ± 0.00	0.00	0.00–0.00
Basophils	%	0, 0, 0, 0, 0, 0, 0, 0, 0, 0, 0, 0, 0, 1	0.07 ± 0.27	0.00	0.00–1.00
Lymphocytes	%	37, 41, 43, 44, 44, 45, 45, 49, 50, 53, 58, 60, 68, 72	50.64 ± 10.37	47.00	37.00–72.00
Monocytes	%	2, 3, 4, 4, 5, 5, 8, 8, 8, 8, 10, 11, 11, 13	7.14 ± 3.37	8.00	2.00–13.00
Thrombocytes	Per 100 WBCs	14, 20, 23, 33, 35, 38, 39, 40, 44, 45, 48, 49, 51, 62	38.64 ± 13.04	39.50	14.00–62.00
Total solid (PP)	g/L	26, 26, 28, 29, 30, 32, 32, 32, 34, 36, 38, 38, 39, 42	33.00 ± 5.02	32.00	26.00–42.00

Farmed NT in Southern Thailand had PCV ranging from 0.23 to 0.45 L/L. Hb concentration ranged from 76 to 119 g/L. The total RBC count ranged from 0.94 to 2.72 × 10¹²/L.

Leucogram analysis showed that total WBC counts ranged from 4.17 to 23.01 × 10^9^/L. Lymphocytes were the predominant WBC type, with absolute numbers ranging from 3.00 to 10.35 × 10^9^/L, followed by neutrophils ranging from 0.71 to 9.66 × 10^9^/L. Monocytes ranged from 0.30 to 2.99 × 10^9^/L, whereas eosinophils and basophils were rare.

## DISCUSSION

### General characteristics of blood cells in NT

Blood cells identified in farmed NT included RBCs, WBCs, and thrombocytes, and were similar to those reported in other teleost species [27, 36]. WBCs were further classified into neutrophils, basophils, eosinophils, lymphocytes, and monocytes. Although heterophils have been reported in some teleost species, such as the armored catfish (*Hoplosternum littorale*) [40], these cells were not observed in NT from Southern Thailand. The authors suggested that the images of all blood cell types obtained in this study could be used to train artificial intelligence models, which might be integrated into automated hematology analyzers to improve the accuracy and precision of piscine hematology analysis, reduce time consumption, and support the implementation of CBC in the aquaculture industry.

### Neutrophils and lymphocytes in NT

Neutrophils in teleost fish act as primary phagocytic cells, migrate to inflammatory sites, and are able to eliminate pathogens through complementary mechanisms [41, 42]. In NT, neutrophils were positive for periodic acid–Schiff (PAS) reaction [43], suggesting that glycogen serves as an important energy source for the phagocytic process. Additionally, NT neutrophils were positive for myeloperoxidase (MPO) staining [43], indicating that this enzyme is likely involved in neutrophil bactericidal activity. Lymphocytes were the most common blood cells in NT, consistent with findings in other teleost species [27, 36, 44].

### Basophils, eosinophils, and monocytes

This study provided the first description of the characteristics of granules in both basophils and eosinophils in Thai NT using Wright’s staining. Despite the use of different staining methods, the eosinophil granule outline observed in this study was similar to that reported previously [43].

Monocytes were present in low numbers in the circulation, accounting for 2%–13% of leukocytes (Table 2). These cells were phagocytic and were considered transient blood cells, as they migrate into connective tissues during inflammation and differentiate into macrophages [45].

### Thrombocytes and their functions

Thrombocytes in fishes show a variety of appearances, including round, oval, spike-shaped, fusiform, or elongated forms [10]. Piscine thrombocytes possess both coagulation functions and phagocytic activity [45].

### Hematological values and comparison with previous studies

The PCV of Thai NT ranged from 23% to 45%, which is higher than the threshold indicating anemia in fish (20%) [34]. Campbell and Grant [34] explained that the normal PCV of many fish species ranges from 20% to 45%, that PCV values greater than 45% are considered indicative of dehydration, and that PCV values lower than 20% are considered indicative of anemia. The PCV may be influenced by intrinsic and extrinsic factors, and normal PCV values in some fish species may be as low as 20%.

The PCV in this study was slightly higher than that reported in Egypt [46], where NT were raised on various dietary ingredients. Another study from Egypt reported PCV values in NT fed diets supplemented with coconut oil, which fell within our preliminary hematologic values [47]. However, both studies [46, 47] reported higher RBC counts than those observed in this study (1.37 ± 0.45 × 10¹²/L).

Total WBC counts in NT (12,860 ± 4,400 cells/µL) were lower than the reference interval for tilapia (21,600–154,700 cells/µL) [31], which was established from mixed groups of fish with different blood collection methods, seasons, ages, and sexes. It is noted that the preliminary hematological values in this study may be significantly limited by the small sample size. Thus, further studies with larger sample sizes are required.

Furthermore, recent studies indicate that hematological parameters in NT are influenced by nutritional and immune-stimulatory factors [48], suggesting that variations may reflect dietary effects rather than solely pre-analytical or technical factors, highlighting the importance of feeding regimes in hematological assessment of farmed fish.

### Methodological considerations and anticoagulant effects

The diluent, Natt and Herrick’s solution, used in this study, is commonly used in piscine hematology [31, 36] as well as in avian hematology [49, 50]. Thus, the lower values observed may have been caused by differences in breed, age, nutrition, or environmental factors.

Corrected WBC counts were performed in this study because the appearance of small WBCs (lymphocytes) and thrombocytes on the hemocytometer was difficult to distinguish. Therefore, all non-RBC cells were counted, and subsequently, corrected WBC counts were calculated using the formula described for species with non-nucleated RBCs [51], with the nucleated RBC number replaced by the thrombocyte number.

Blood smears of fish in Group II had several karyorrhectic (apoptotic) cells and smudge (basket) cells (Figures 1B and 1C), suggesting blood cell damage. These artifacts may have been induced by prolonged-storage in EDTA, as described previously [52, 53]. Previous studies have also indicated erythrocyte swelling associated with EDTA in some teleost species [32], whereas heparin may better preserve cellular morphology in certain species. Thus, heparin should be considered the anticoagulant of choice for piscine blood analysis. To standardize and improve laboratory management in piscine hematology, further investigations into the effects of storage conditions are recommended.

### Blast cells and possible physiological stress

Furthermore, several blast cells (Figure 1A) were found in the market-transported fish (Group I). The authors assumed that the increased number of blast cells or transformed mature cells may have been related to physiological changes occurring during transportation, during which several fish were kept in the same tank. Although NT subjected to hypoxic stress have been reported to show no significant changes in blood biochemistry [54], the lineage and underlying cause of these blast cells require confirmation.

### Observation of Anaplasmataceae-like organism

Although blood parasites were not within the scope of this study, the authors observed an *Anaplasmataceae*-like organism. A previous study reported *Anaplasmataceae* infection in NT from Brazil [17]. This study may represent the first potential report of an emerging *Anaplasmataceae*-like organism in Thai NT, providing baseline information for future studies. The authors suggested that further investigations using both molecular techniques and transmission electron microscopy are required to confirm *Anaplasmataceae* infection in NT in Thailand.

## CONCLUSION

This study provides a comprehensive preliminary characterization of hematological profiles and blood cell morphology in farmed sex-reversed male NT from Southern Thailand. The results demonstrated that erythrocytes, leukocytes, and thrombocytes exhibited morphological features consistent with those reported in other teleost species, with lymphocytes representing the predominant leukocyte population, followed by neutrophils, while eosinophils and basophils were rarely observed. The hematological values obtained, including PCV, Hb concentration, and leukocyte distribution, fell within biologically plausible ranges, although some variations were noted when compared with previously published data, likely due to environmental, nutritional, and methodological differences. Additionally, the detection of an *Anaplasmataceae*-like inclusion body highlights the potential presence of emerging hemoparasites in farmed populations, warranting further investigation.

From a practical perspective, the findings of this study provide essential baseline hematological data that can support routine health assessment, disease diagnosis, and monitoring of physiological status in NT aquaculture systems. The detailed morphological descriptions and high-quality cytological observations can enhance laboratory competency, particularly in manual CBC evaluations, which remain the standard approach in fish hematology due to nucleated blood cells. Furthermore, these data may serve as a foundation for developing artificial intelligence-assisted diagnostic tools and improving the accuracy and efficiency of hematological analyses in aquaculture settings.

A key strength of this study lies in the integration of both qualitative and quantitative hematological assessments, including detailed morphometric analysis of blood cells and strict inclusion criteria for sample quality, which ensured reliable interpretation of results. The use of standardized staining techniques and blinded evaluation by trained personnel further strengthens the validity of the findings. However, several limitations should be acknowledged. The relatively small sample size (n = 14) restricts the establishment of definitive reference intervals, and the absence of water quality data limits the ability to correlate environmental factors with hematological variations. In addition, the lack of molecular confirmation for the observed *Anaplasmataceae*-like organism prevents definitive identification of potential infections.

Future research should focus on expanding sample size to establish robust reference intervals, incorporating seasonal and environmental variations, and evaluating the effects of nutrition, management practices, and stress on hematological parameters. Molecular and ultrastructural investigations are also required to confirm the presence and epidemiological significance of hemoparasites in NT populations. Moreover, comparative studies evaluating different anticoagulants and storage conditions would further improve methodological standardization in piscine hematology.

In conclusion, this study contributes valuable baseline data on blood cell morphology and hematological parameters in farmed NT, supporting improved diagnostic capability, enhanced laboratory standardization, and informed health management practices. These findings ultimately contribute to the sustainability and productivity of aquaculture systems and reinforce the role of hematological assessment in ensuring fish health and food security.

## DATA AVAILABILITY

The data generated during the study are included in the manuscript.

## AUTHORS’ CONTRIBUTIONS

KS and PP: Conceptualization and Methodology. DP, KS, and PP: Investigation and data collection. KS: Writing-original draft preparation. DP and PP: Sample collection, data curation, and funding. PP: Software and validation. KS, PP, TNT, and WL: Data analysis. PP, TNT, and WL: Data visualization and writing-review and editing. All authors have read and approved the final version of the manuscript.
